# Primary epidural malignant hemangiopericytoma of thoracic spinal column causing cord compression: case report

**DOI:** 10.1590/S1516-31802004000500009

**Published:** 2004-09-01

**Authors:** Mohammad Mohammadianpanah, Simin Torabinejad, Mohammad Hadi Bagheri, Shapour Omidvari, Ahmad Mosalaei, Niloofar Ahmadloo

**Keywords:** Hemangiopericytoma, Thoracic verte-brae, Neoplasms, Nerve crush, Laminectomy, Hemangiopericitoma, Vértebras torácicas, Neoplasias, Compressão nervosa, Laminectomia

## Abstract

**CONTEXT:**

Hemangiopericytoma is an uncommon mesenchymal neoplasm that rarely affects the spinal canal. Primary malignant hemangiopericytoma of the spinal column is extremely rare.

**CASE REPORT:**

We report on a case of primary epidural malignant hemangiopericytoma of the thoracic spinal column that invaded vertebral bone and caused spinal cord compression in a 21-year-old man. The patient presented with progressive back pain over a four-month period that progressed to paraparesis, bilateral leg paresthesia and urinary incontinence. The surgical intervention involved laminectomy and subtotal resection of the tumor, with posterior vertebral fixation. Postoperative involved-field radiotherapy was administered. A marked neurological improvement was subsequently observed. We describe the clinical, radiological, and histological features of this tumor and review the literature.

## RESUMO

**CONTEXTO:**

Hemangiopericitoma é uma neoplasia mensequimal incomum que raramente afeta o canal espinhal. O hemangiopericitoma maligno primário da coluna vertebral é extremamente raro.

**RELATO DE CASO:**

Relatamos um caso de hemangiopericitoma maligno epidural primário da coluna vertebral torácica que invade o osso vertebral, causando a compressão do cordão espinhal em um homem de 21 anos. O paciente apresentou-se com dor progressiva nas costas durante quatro meses, que progrediu para paraparesia, parestesia bilateral do pé e incontinência urinária. A intervenção cirúrgica com laminectomia e ressecção do tumor e fixação posterior foi executada. A radioterapia pós-operatória no campo envolvido foi realizada. Marcante melhoria neurológica foi observada subseqüentemente. Descrevemos as características clínicas, radiológicas, e histológicas deste tumor e revemos a literatura.

## INTRODUCTION

Hemangiopericytoma is a rare vascular tumor originating from Zimmerman's pericytes, which are contractile spindle cells surrounding the capillaries and postcapillary venules. This neoplasm accounts for less than 1% of all vascular tumors.^1^ It may be found throughout the body, but the most common location is the deep soft tissues.^2^ The most common sites for soft tissue hemangiopericytoma are the lower extremities, followed by the retroperitoneum and the head and neck region.^3^

Such tumors occur in both sexes with equal frequency and are found mainly in adults and rarely in children.^4^ They occur most often in the fifth and sixth decades of life in the soft tissue form and the fourth and fifth decades in the osseous form, with a slight predominance of osseous hemangiopericytoma among males.^5^

The clinical presentation of hemangiopericytoma varies depending on the tumor size and the disease site. Pain may be a late symptom in soft tissue hemangiopericytoma. Conversely, in osseous hemangiopericytoma, pain and swelling are the most common complaints.

These tumors can be locally aggressive, recur locally, and even metastasize. The lungs and bone are the most common sites for metastasis.^3^ The radiographic features of these tumors are nonspecific. However, characteristic angiographic features may aid in the diagnosis. A “spider-shaped” appearance in the arterial phase, and dense, well-demarcated, round or oval tumor staining in the venous phase, are the characteristic angiographic findings from hemangiopericytoma.

Adequate surgical resection, when feasible, is the first choice of treatment in all cases of hemangiopericytoma. Preoperative endovascular embolization is recommended because of the pronounced tendency to hemorrhage throughout biopsy and surgical procedures.

In cases of benign hemangiopericytoma, complete surgical resection is sufficient. On the other hand, in cases of malignant hemangiopericytoma, the addition of radiation therapy or chemotherapy may be indicated, especially in cases with high-grade lesions, large tumors, or resection with positive surgical margins. Radiotherapy alone is only indicated for unresectable tumors. Likewise, chemotherapy is also used for treatment of unresectable tumors and metastatic disease.

These tumors usually progress to a favorable outcome, but 20%-30% of cases behave in a malignant fashion, as in our case. It needs to be considered that malignant cases may consist of aggressive neoplasm with a high rate of local recurrence and a propensity to metastasize.^2,6^

## CASE REPORT

### Clinical findings

A 21-year-old man with a four-month history of progressive back pain that was followed by lower extremity weakness and urinary incontinence, was admitted to the neurosurgery department. Neurological examination at the time of referral found a strength score of 2/5 bilaterally in his lower extremities, a sensory level of T5, hypotonia and increased deep tendon reflexes in the lower limbs, and impaired proprioceptive sensation. Urinary incontinence and tenderness of the upper thoracic spine was also present.

### Radiological findings

**Figure 1:**
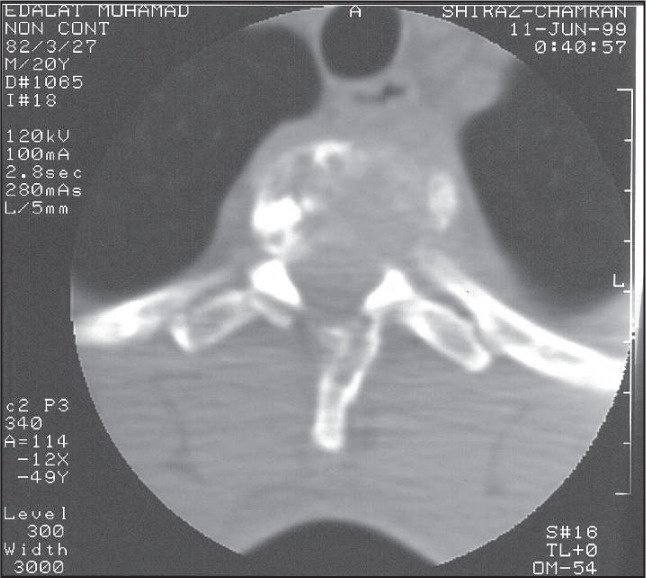
Axial computed tomography scan of the chest showing the expansion and destruction of the T2 vertebral body, with soft tissue extending to the spinal canal in a young man with hemangiopericytoma.

Plain x-ray of the thoracic spine revealed collapse of the body of the second thoracic spine vertebra. Computed tomography (CT) scan of this region revealed destruction of the body, left pedicle, and left lamina of the T2 thoracic spine vertebra, associated with a soft tissue mass extending to the spinal canal around the thecal sac. Paravertebral extension was also evident (Figure 1).

**Figure 2:**
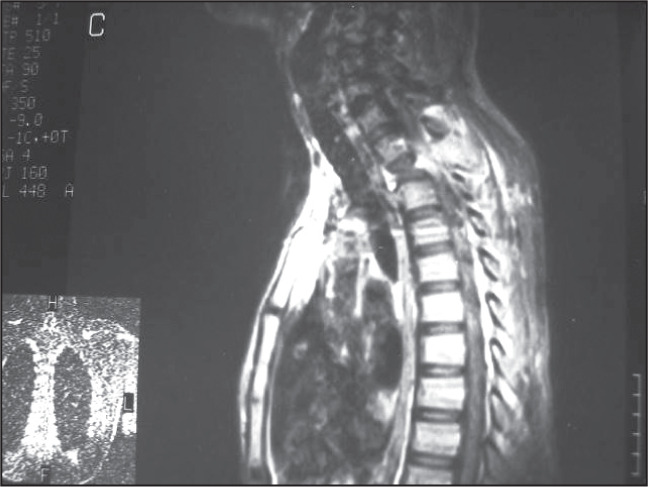
Sagittal T1-weighted gadolinium-enhanced magnetic resonance image showing compression fracture of the T2 vertebral body with severe spinal cord compression, in a young man with hemangiopericytoma.

Magnetic resonance (MR) images showed an infiltrating mass involving the vertebral body of the second thoracic spine vertebra, with compression fracture of the T2 vertebral body causing severe spinal cord compression (Figure 2).

**Figure 3:**
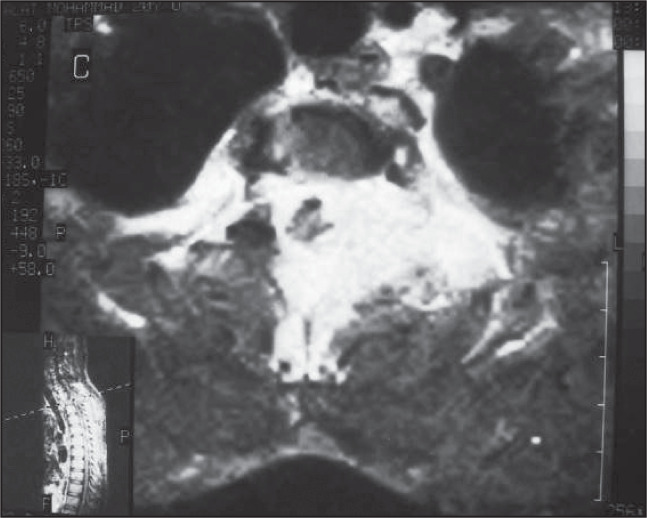
Axial T1-weighted gadolinium-enhanced magnetic resonance image showing dense enhanced paravertebral mass encasing the spinal canal, suggestive of hemangiopericytoma in a young man.

The tumor was hyperintense in relation to the surrounding muscle in the T1 and T2 weighted images, with dense enhancement after gadolinium injection. Encasement of the spinal canal, of paraspinal extent, was also shown (Figure 3).

The radiological differential diagnoses included metastases, solitary plasmacytoma, leukemia, lymphoma, and tuberculosis infection.

### Treatment

The patient underwent an emergency operation, with the initial diagnosis of a malignant tumor causing pathological fracturing of the T2 vertebral body. Bilateral laminectomy, left side facetotomy of the second thoracic spine vertebra and subtotal resection of the epidural mass were performed. Posterior fixation of C7-T5 was performed using a sublaminar hook. En bloc resection was not feasible at that time, because of the encasement of the spinal cord by the tumor.

Postoperative involved-field radiotherapy with a total dose of 46 Gy was delivered. Because of the incomplete surgical resection of the tumor, and the high risk of locoregional and distant recurrence, adjuvant chemo-therapy was considered.

### Pathological findings

A paravertebral mass measuring 5 × 5 × 1 cm with a smooth rubbery surface was seen. The cut surface showed brown to red areas with multiple hemorrhagic foci. Multiple fragments of bony tissue were also identified.

**Figure 4:**
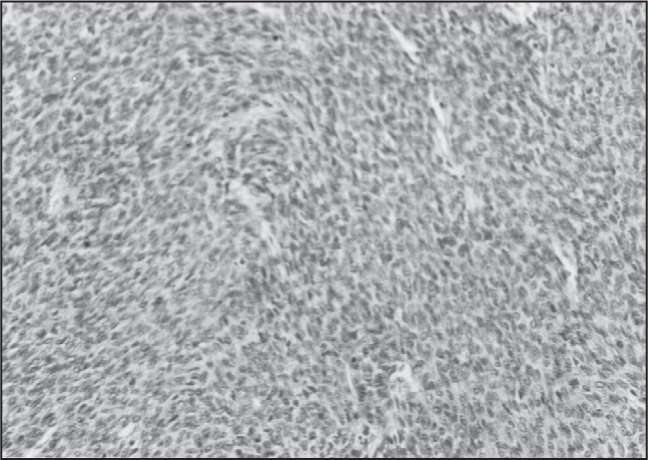
High-power view of the epidural thoracic spinal column hemangiopericytoma (hematoxylin-eosin, 400 x).

Optical microscopy examination of hematoxylin-eosin stained tissue sections demonstrated hemangiopericytoma. The tumor cells were arranged in sheets and fascicles with intervening staghorn-shaped blood vessels. The individual tumor cells had round to oval hyperchromatic nuclei with modest amounts of indistinct eosinophilic cytoplasm at the cell borders. Multiple areas of necrosis were also seen. There were frequent mitotic figures and apoptotic cells. Tumoral tissue permeated between bone trabeculae (Figure 4).

## DISCUSSION

Hemangiopericytoma is regarded as a less organoid type of glomus tumor, arising from Zimmerman's pericytes. Stout and Murray first described this uncommon tumor in 1942.^1^ The tumor occurs primarily in adults and is often in deep-seated soft tissues. Benign and malignant forms of hemangiopericytoma have been described.^3,4^

The natural history of hemangiopericytoma correlates well with cellularity, mitotic activity, anaplasia, necrosis, and hemorrhage. Four or more mitoses per high-power microscopy field, necrotic foci and increased cellularity are suggestive of malignancy and poor prognosis. Local recurrence is an ominous sign, and often heralds the appearance of distant metastases, most probably in the lungs and bone.^3^

These tumors are radiographically non-specific, whether via plain x-ray, CT scan or MR imaging, and they usually appear as well-encapsulated soft tissue masses. Their angiographic features may aid in the diagnosis. The primary goal in diagnosing a primary hemangiopericytoma of the spinal column is to rule out the possibility that the tumor represents metastasis from other primary sites.

Just as in cases of soft tissue hemangiopericytoma, surgical resection remains the mainstay of therapy for osseous hemangiopericytoma. Spinal hemangiopericytoma should ideally be resected en bloc, so as to reduce intraoperative bleeding and local recurrence. However, radical resection is frequently impossible without significantly compromising limb function or causing neurological deficits.^6^ Although the value of adjuvant chemo-therapy or radiation therapy is still uncertain, these types of adjuvant therapy should be considered in cases with high-grade, unresectable or recurrent disease. The prognosis of these tumors correlates with the resectability and histological grading of the tumor.

## CONCLUSIONS

This article describes an extremely rare case of primary epidural malignant hemangiopericytoma of the thoracic spinal column that invaded the vertebral bone and caused spinal cord compression in a 21-year-old man.

The clinical, radiological, and pathological features of this tumor have been described and the differential diagnoses for hemangiopericytoma have been discussed. Hemangiopericytoma of the spinal column should be considered potentially malignant and therefore be treated aggressively.^6^

In general, surgery always is the treatment of choice. Radiotherapy and/or chemotherapy may be indicated as primary or adjuvant treatment, depending on the tumor resectability and aggressiveness. In high-risk patients such as our case, the risk of local and distant recurrence should be anticipated, and therefore prolonged follow-up is strongly recommended.
